# Frequency and Early Predictors of Cognitive Deterioration in Amyotrophic Lateral Sclerosis: A Longitudinal Population‐Based Study

**DOI:** 10.1002/ana.27194

**Published:** 2025-02-01

**Authors:** Barbara Iazzolino, Francesca Palumbo, Cristina Moglia, Umberto Manera, Maurizio Grassano, Enrico Matteoni, Sara Cabras, Maura Brunetti, Rosario Vasta, Marco Pagani, Gabriele Mora, Antonio Canosa, Andrea Calvo, Adriano Chiò

**Affiliations:** ^1^ ALS Center, ‘Rita Levi Montalcini’ Department of Neuroscience University of Torino Turin Italy; ^2^ Division of Neurology 1 Azienda Ospedaliero‐Universitaria Città della Salute e della Scienza of Torino Turin Italy; ^3^ Institute of Cognitive Sciences and Technologies National Research Council Rome Italy

## Abstract

**Objective:**

The objective is to evaluate cognitive and behavioral progression and identify early predictors of these changes in a cohort of amyotrophic lateral sclerosis (ALS) patients.

**Methods:**

A total of 161 ALS patients were tested at diagnosis (T0), and 107 were re‐tested after 1 year (T1) using cognitive/behavioral tests. All patients underwent whole‐genome sequencing, and 46 patients (ALS‐normal cognition [CN]) underwent [18F]Fluorodeoxyglucose positron emission tomography.

**Results:**

Of the 161 patients, 107 were re‐rested at T1; non‐retested patients included 10 with frontotemporal dementia and 44 who were either non‐testable or deceased. At T0, 67 patients (62.6%) were classified as ALS‐CN, whereas 40 (38.4%) showed some degree of cognitive/behavioral impairment. Eighteen ALS‐CN patients (26.9%) experienced cognitive decline at T1. Phenoconverters had lower baseline scores in letter fluency (Letter Fluency Test [FAS]) (*p* < 0.001), Edinburgh Cognitive and Behavioral ALS Screen (ECAS) verbal fluency score (*p* = 0.017). Both tests were independently predictive of phenoconversion in binary logistic regression models, with optimal cut‐off scores of 28.75 and 14.2, with good sensitivity and specificity. Other predictors included older age, lower education, and ALS‐related genetic variants. Phenoconverters were hypometabolic in the left temporal lobe. Thirteen (32.5%) of the 40 patients with cognitive impairment at T0 worsened by T1, with FAS (*p* = 0.02) and the ECAS verbal fluency score (*p* = 0.023) predicting further decline.

**Interpretation:**

Approximately 30% of ALS patients experienced cognitive/behavioral decline within the first year after diagnosis. FAS and ECAS verbal fluency were predictive of cognitive phenoconversion. Our findings highlight the importance of early detection of at‐risk individuals and the need for longitudinal cognitive assessments to monitor disease progression. ANN NEUROL 2025;97:1122–1133

Amyotrophic lateral sclerosis (ALS) is a progressive degenerative disorder primarily involving the motor function at upper and lower motor neuron level. Clinical and population based‐studies have shown that approximately 50% of ALS patients exhibit some level of cognitive decline at the time of diagnosis,^1^, ^2^, ^3^ ranging from mild degrees of cognitive impairment to frontotemporal dementia (FTD).^4^ The most frequently impaired cognitive domains are executive function, language, and social cognition.^2^, ^5^ ALS patients with comorbid cognitive and behavioral impairment may have more rapid decline of motor function^6^, ^7^ and poorer prognosis.^2^, ^3^

Cognitive dysfunction in ALS has been found to correlate with various factors, including age,^8^, ^9^ disease staging,^7^, ^10^, ^11^, ^12^ bulbar involvement,^13^, ^14^, ^15^ presence of genetic variants, in particular *C9ORF72*,^14^, ^16^, ^17^ and cognitive reserve.^18^, ^19^

Despite significant progress in understanding cognitive and behavioral impairment in ALS, it remains unclear whether and how these functions deteriorate over time. The limited longitudinal studies conducted to date have produced conflicting results.^6^, ^9^, ^17^, ^20^, ^21^, ^22^ In general, it is widely acknowledged that patients who already exhibit cognitive impairment at their first examination may experience further decline over time.^6^, ^9^ However, the likelihood of cognitively normal patients deteriorating over time is less clear. Some studies suggest that this does not occur,^6^, ^9^, ^17^ whereas others report that a variable percentage of cognitively normal patients show deterioration during the follow‐up period.^20^, ^21^, ^22^ Most of these studies, however, are limited by high attrition rates and the use of a reduced battery of tests. Moreover, no studies have specifically examined whether certain cognitive functions or tests at diagnosis can predict future decline.

This study aims are to evaluate the progression of cognitive and behavioral functions over time and to identify early predictors of these changes in a population‐based cohort of ALS patients.

## Methods

A total of 161 ALS patients, consecutively seen between 2017 and 2019 at the Turin ALS Center and identified through the Piemonte and Valle d'Aosta Register of ALS (PARALS), a prospective epidemiologic register that covers 2 Italian regions,^23^ were recruited for this study. Patients were eligible if they had a diagnosis of ALS according to the Gold Coast criteria.^24^ At the time of cognitive testing, the ALS Functional Rating Scale‐Revised (ALSFRSr) score and King's and Milano‐Torino (MiToS) staging were recorded.

Patients with a history of disorders that may potentially affect cognition (ie, mental retardation, major stroke, and severe head injuries), alcohol or drug dependence, severe mental illness, or use of high‐dose psychoactive medications were excluded from the study. Patients who were not of native Italian language were assessed only through an unstructured interview and were excluded from the analysis. Patients were tested within 2 month from diagnosis (T0) and re‐tested after 1 year (±3 months) (T1). Patients with a diagnosis of ALS‐FTD at the T0 evaluation were excluded from the follow‐up study. ALSFRSr mean monthly decline (∆ALSFRSr) was calculated using the following formula: (48 − ALSFRSr total score at time of testing)/(months from onset to time of testing). A total of 132 age‐ and sex‐matched controls (HC), recruited among residents in retirement homes or non‐consanguineous relatives of cases, were also tested with the same battery.

### Neuropsychological Assessment and Domain Classification of Tests

Patients and HC underwent a battery of neuropsychological tests encompassing executive function, verbal and visual memory, attention and working memory, visuospatial function, language, social cognition, and behavior. The battery of tests was administered within 2 months from diagnosis. The tests were selected according to the Diagnostic Criteria for the Behavioral variant of Frontotemporal Dementia,^25^ and ALS‐FTD Consensus Criteria (ALSFTD‐CC).^26^ The list of tests and their classification according to the main explored neuropsychological domain are reported in the Supplementary Methods and Table S1.^5^, ^27^, ^28^ The raw scores of all tests were age‐, sex‐, and education‐corrected using the more recent published Italian normative (Table S2). Additionally, normal cut‐off was based on Italian normative. For the 2 tests that appeared to predict cognitive phenoconversion (Letter Fluency Test [FAS], and the verbal fluency domain of The Edinburgh Cognitive and Behavioral ALS Screen [ECAS]) we also considered the cut‐off obtained with our cohort of HCs, calculated as mean – 2 standard deviations (SD).

Anxiety and depression were assessed with the Hospital Anxiety and Depression Scale (HADS); the item “I feel slowed down” patients were instructed not to refer to physical disability.^3^ The average time for performing the whole battery was 90 minutes. In a few cases, the battery was completed within 1 week during a second session.

According to the ALSFTD‐CC^26^ patients were classified into 5 cognitive categories: (1) patients with normal cognition (ALS‐CN); (2) patients with isolated cognitive impairment (ALSci), that is, patients with evidence of executive and/or language dysfunction (executive impairment was defined as impaired verbal fluency [letter] and/or impairment on 2 other non‐overlapping measures of executive functions); (3) patients with isolated behavioral impairment (ALSbi), characterized by apathy with or without other behavioral changes; (4) patients with both cognitive and behavioral impairment (ALScbi), meeting the criteria for both ALSci and ALSbi; and (5) patients with frontotemporal dementia (ALS‐FTD).

### Genetic Screening

All patients of this cohort underwent whole genome sequencing (WGS). WGS methodology and quality control filters are reported in detail elsewhere.^29^ Pathogenetic variants of 46 ALS‐related genes were extracted.^29^ Variants classification was based on the 2015 American College of Medical Genetics and Genomics–Association for Molecular Pathology (ACMG‐AMP) guidelines.^30^ All patients were screened by a repeat‐primed polymerase chain reaction assay for the presence of the GGGGCC hexanucleotide expansion in the first intron of *C9ORF72* (Renton et al,^31^). Repeat lengths of 30 or more units with the characteristic sawtooth pattern were considered to be pathogenic.^31^

### Positron Emission Tomography Acquisition and Preprocessing

Brain 2‐[18F]fluorodeoxyglucose positron emission tomography ([18F]FDG‐PET) was available for 46 patients of the cohort, that is, 26 ALS‐CN patients at T0 (6 phenoconverters and 20 non‐phenoconverters) and 20 cognitively impaired patients (8 who showed further progression and 12 who remained stable). Brain 2‐[18F]FDG‐PET was performed according to published guidelines.^32^ Patients fasted for at least 6 hours before the exam. Blood glucose was <7.2mmol/l in all cases before the procedure. After a 20‐minute rest, about 185MBq of [18F]FDG was injected. The data acquisition started 60 minutes after the injection. PET/computed tomography (CT) scans were performed on a Discovery ST‐E System (General Electric, Boston, MA). Brain CT and PET scan were sequentially acquired, and the former was used for attenuation correction of PET data. The PET images were reconstructed with 4 iterations and 28 subsets with an initial voxel size of 2.34 × 2.34 × 2.00mm, and data were collected in 128 × 128 matrices. Images were spatially normalized to a customized brain 2‐[18F]FDG‐PET template^33^ and subsequently smoothed with a 10‐mm filter in MATLAB R2018b (The MathWorks, Natick, MA). Intensity normalization was performed at individual level averaging each voxel for the mean value of the whole brain.

### PET Statistical Analysis

First, we focused on ALS‐CN and we used the analysis of covariance (ANCOVA) of SPM12 for the comparison of cognitive/behavioral converters and non‐converters, including age at PET, sex, type of onset (bulbar vs. spinal), King's stage at time of PET and *C9ORF72* status as covariates. Second, we performed an analogue analysis comparing patients who did not worsen from T0 to T1 and patients who did, pooling all cognitive categories. The height threshold for both analyses was set at *p* < 0.001 (*p* < 0.05 false discovery rate‐corrected at cluster level). Only clusters containing greater than 125 contiguous voxels were considered significant. Brodmann areas were identified at a 0 to 2‐mm range from the Talairach coordinates of the SPM output isocenters corrected by Talairach Client (http://www.talairach.org/index.html).

### Statistical Methods

Comparisons between tests were conducted on age‐, sex‐, and education‐corrected scores. Because of non‐normal distribution in some cognitive test scores, the Mann–Whitney *U* test was used for comparisons. Two‐tailed *p*‐values are reported and Holmes correction for multiple testing was used. Binary logistic regression was performed to determine the variables, which independently were related to phenoconversion. The following variables were included in the models: age at time of testing, sex, site of onset (bulbar vs. spinal), years of education, time from disease onset to testing, ALSFRSr score at time of testing, presence or absence of a genetic variants, King's and MiToS staging. All analyses were carried out with SPSS 29.0 statistical package (SPSS, Chicago, IL).

### Standard Protocol Approvals, Registrations, and Patient Consents

The study was approved by the Ethics Committee of the ALS Expert Center of Torino (Comitato Etico Azienda Ospedaliero‐Universitaria Città della Salute e della Scienza, Torino, 0036344, 0038876, and 0064510). Patients provided written informed consent before enrollment. The databases were anonymized according to Italian law for the protection of privacy.

## Results

A total of 161 ALS patients consecutively seen in the Turin ALS center between 2017 and 2019 and included in the PARALS were recruited. Of these, 10 were not re‐tested because had already ALS‐FTD and 44 were not testable or were deceased at T1. The remaining 107 patients were re‐tested after a median time of 359 days (interquartile range [IQR]: 252–419 days). In Table 1, we have compared the characteristics at T0 of the 107 patients who were re‐tested and of the 54 patients who were not re‐tested. They differ for age at onset, ALSFRSr score, and King's staging. They had also a shorter survival from T0 (Fig S1) (median survival, re‐tested patients 2.31 years (IQR: 1.30–4.14), non‐re‐tested patients 1.61 years (IQR: 0.80–2.56)). A comparison of the cognitive classification at T0 of the originally recruited patients is reported in Table S3. The final study population comprised 107 patients (66.5% of the originally enrolled cohort) of whom 67 (62.6%) were classified as ALS‐CN, 22 (20.6%) as ALSci, 12 (11.2%) as ALSbi, and 6 (5.6%) as ALScbi.

**TABLE 1:** Comparison of ALS Patients Enrolled in the Study Who Were and Were Not Reassessed at T1

	Reassessed patients (n = 107)	Non‐reassessed patients (n = 54)	Patients originally enrolled (n = 161)	*p* (reassessed vs non‐reassessed patients)
Sex (M, %)	64 (59.8)	30 (55.6)	94 (58.4)	0.62
Mean age at T0 (yr, SD)	65.3 (10.1)	68.7 (10.7)	66.6 (10.5)	0.032
Mean education (yr, SD)	9.4 (4.1)	9.5 (4.5)	9.4 (4.3)	0.90
Mean time from onset to test (mo, SD)	14.1 (5.3)	12.2 (5.6)	13.5 (6.2)	0.021
Site of onset (bulbar, %)	35 (32.7)	22 (40.7)	57 (35.4)	0.38
Mean ALSFRSr score (points, SD)	40.6 (5.1)	37.8 (6.9)	49.66 (6.3)	0.004
King's staging (1/2/3/4)	49/35/21/2	14/15/21/4	63/50/42/6	0.001
MiToS staging (0/1/2)	92/13/2	43/9/2	125/22/4	0.55

Cognitive classification is reported in Table S4.

ALS = amyotrophic lateral sclerosis; ALSFRSr = ALS Functional Rating Scale–Revised; M = male; MiToS = Milano‐Torino staging; SD = standard deviation.

A total of 30 patients (28.0%) carried a pathogenic variant of an ALS‐related gene, with the most common being the *C9ORF72* GGGGCC hexanucleotide repeat, found in 8 cases (7.5%). A complete list of genes, along with the corresponding cognitive classification of patients, is provided in Table S4.

### ALS‐CN Phenoconverters

**FIGURE 1:**
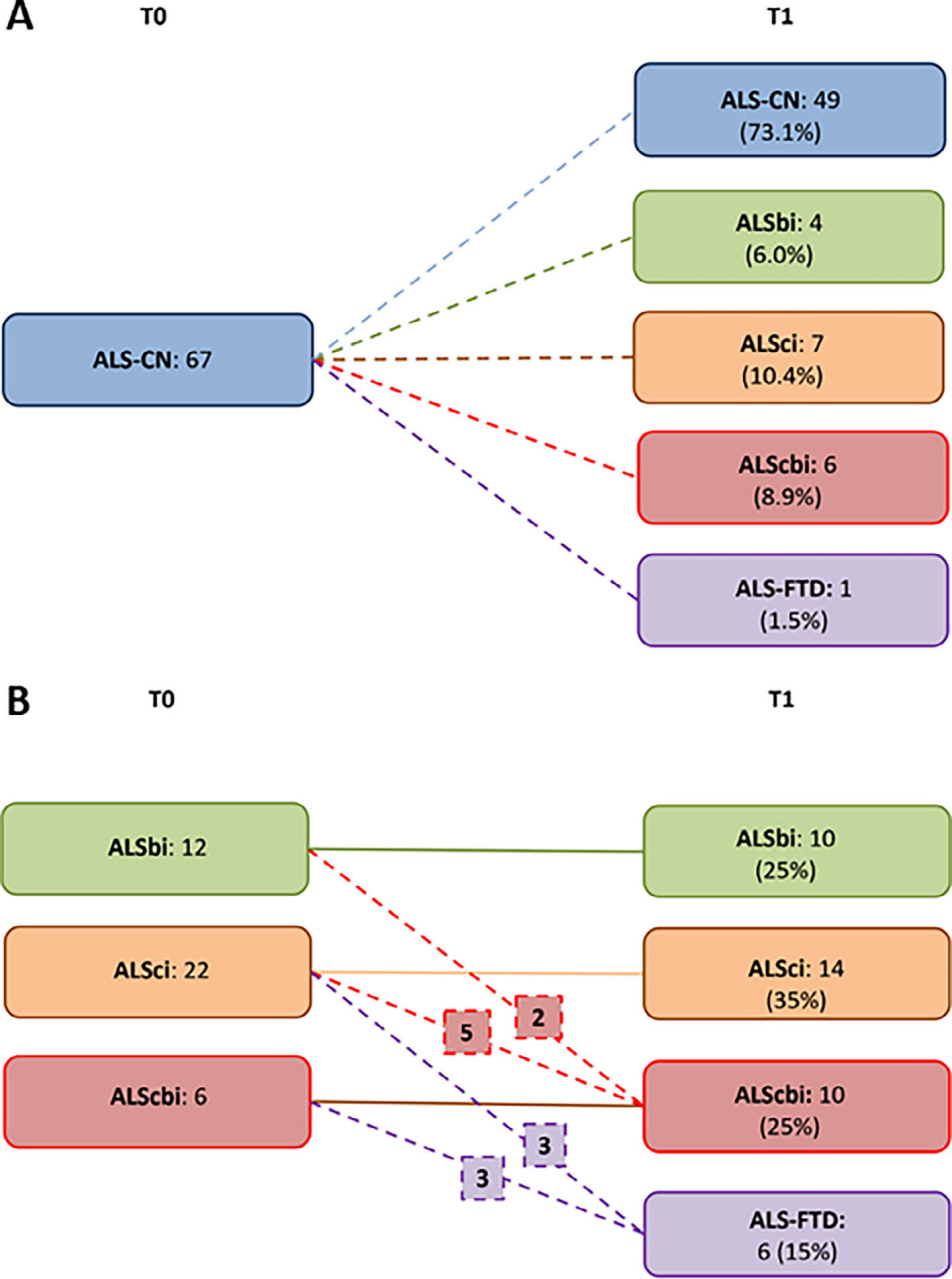
(A) ALS‐CN patients at T0 and their cognitive and behavioral diagnosis at T1. (B) ALSbi, ALSci, and ALScbi patients at T0 and their cognitive and behavioral diagnosis at T1. ALS = amyotrophic lateral sclerosis; ALS‐CN = patients with normal cognition; ALSci = patients with isolated cognitive impairment; ALSbi = patients with isolated behavioral impairment; ALScbi = patients with both cognitive and behavioral impairment.

Eighteen (26.9%) of the 67 ALS‐CN patients showed a phenoconversion to either ALSci (7 cases), ALSbi (4 cases), ALScbi (6 cases), or ALS‐FTD (1 case) (Fig 1A). The comparison of clinical characteristics of patients who had a worsening of cognitive/behavioral status and those who did not is reported in Table 2. The only significant difference between the 2 groups was a higher age at time of testing (69.1 years [SD: 5.8] vs. 62.3 years [SD: 10.1], *p* = 0.009). Comparing the cognitive test scores at T0 among ALS‐CN, phenoconverters had significant lower scores in FAS (*p* < 0.001), Rey Auditory Verbal Learning Test, immediate recall (RAVL‐IR) (*p* = 0.019), Rey‐Osterrieth Complex Figure Test, immediate recall (ROCF‐IR) (*p* = 0.043), Raven's Colored Progressive Matrices (CPM47) (*p* = 0.021) and ECAS verbal fluency score (*p* = 0.017) (Table S5). However, none of the lower test scores in phenoconverters were below 2 SD from the scores of HCs locally tested. Line graphs showing individual changes in FAS and ECAS verbal fluency scores between T0 and T1 are presented in Figure S3. Overall, the FAS score decreased by 10.3% (95% CI, 8.7–11.9%), with 45 patients experiencing a decline, 5 showing no change, and 16 showing an increase. Notably, the median increase in the FAS score among the latter group was 4, indicating a relatively limited improvement. Similarly, the ECAS verbal fluency score decreased by 9.4% (95% CI, 8.4–10.2%), with 40 patients showing a decline, 7 showing no change, and 20 demonstrating an increase (median: 1.5 points).

**TABLE 2:** Comparison of Characteristics at T0 of CN‐ALS Patients Who Had a Cognitive/Behavioral Phenoconversion at T1 and Those Who Did Not Phenoconverted

	ALS‐CN phenoconverters (n = 18)	ALS‐CN non‐phenoconverters (n = 49)	*p*
Mean age at testing (yr, SD)	69.1 (5.8)	62.3 (10.1)	0.009
Mean time from onset to testing (mo, SD)	12.1 (7.9)	11.8 (8.3)	0.76
Mean education (yr, SD)	9.2 (4.0)	10.2 (4.2)	0.49
Sex (M, %)	9 (50)	30 (61.2)	0.29
Site of onset (bulbar, %)	7 (38.9)	13 (26.5)	0.42
Carriers of genetic variants (yes, %)	8 (44.4)	11 (22.4)	0.07
Mean ALSFRSr score at T0 (SD)	40.2 (5.1)	41.4 (4.7)	0.35
Mean ALSFRSr decline (points/mo) from onset to T0	0.64 (0.45)	0.64 (.51)	0.81
King's staging at T0 (1/2/3/4)	7/6/4/1	29/10/10/0	0.20
MiToS staging at T0 (0/1/2)	17/0/1	45/3/1	0.44

ALS = amyotrophic lateral sclerosis; ALS‐CN = ALS patients with normal cognition; ALSFRSr = ALS Functional Rating Scale–Revised; M = male; MiToS = Milano‐Torino staging; SD = standard deviation.

Regarding the behavioral component, at T0 no ALS‐CN patient had pathological apathy or dysexecutive subscores at frontal systems behavior scale (FrSBe), and only 1 had a pathological disinhibition subscore. At T1, 10 had pathological subscores for apathy, 11 for disinhibition, and 6 for executive dysfunction. No patient had a pathological ECAS behavioral score at T0, whereas 10 had a pathological score at T1.

To better define the characteristics of the subgroup of ALS‐CN patients who phenoconverted, we performed 2 separate binary logistic regression models to find if other factors besides FAS and ECAS verbal fluency score were related to phenoconversion. We performed 2 separate models because of the high collinearity of the 2 tests. In the model including FAS, the factors independently related to phenoconversion were FAS, age at time of testing, and the presence of an ALS‐related genetic variant (Table 3). In the model including ECAS verbal fluency, the factors independently related to worsening of cognitive status were ECAS verbal fluency score, age at time of testing, years of education, and the presence of an ALS‐related genetic variant (Table 3).

**TABLE 3:** Factors Independently Related to Phenoconversion: Binary Logistic Regression Models

Factors	OR (95% CI)	*p*
Model including FAS		
FAS	0.893 (0.815–0.978)	0.015
Age at time of testing	1.124 (1.022–1.237)	0.016
Presence of an ALS‐related genetic variant	0.86 (1.207–21.43)	0.027
Model including ECAS verbal fluency score		
ECAS verbal fluency score	0.804 (0.66–0.979)	0.030
Age at time of testing	1.012 (1.001–1.022)	0.026
Yr of education	0.482 (0.246–0.942)	0.033
Presence of an ALS‐related genetic variant	5.187 (1.231–21.86)	0.025

Only significant factors are reported. The following variables were included in the models: age at time of testing, sex, site of onset (bulbar vs spinal), years of education, time from disease onset to testing, ALSFRSr score at time of testing, presence or absence of a genetic variants, King's and MiToS staging.

ALS = amyotrophic lateral sclerosis; ALSFRSr = ALS Functional Rating Scale–Revised; CI = confidence interval; ECAS = Edinburgh Cognitive and Behavioural ALS Screen; FAS = Letter Fluency Test; MiToS = Milano‐Torino staging; OR = odds ratio.

**FIGURE 2:**
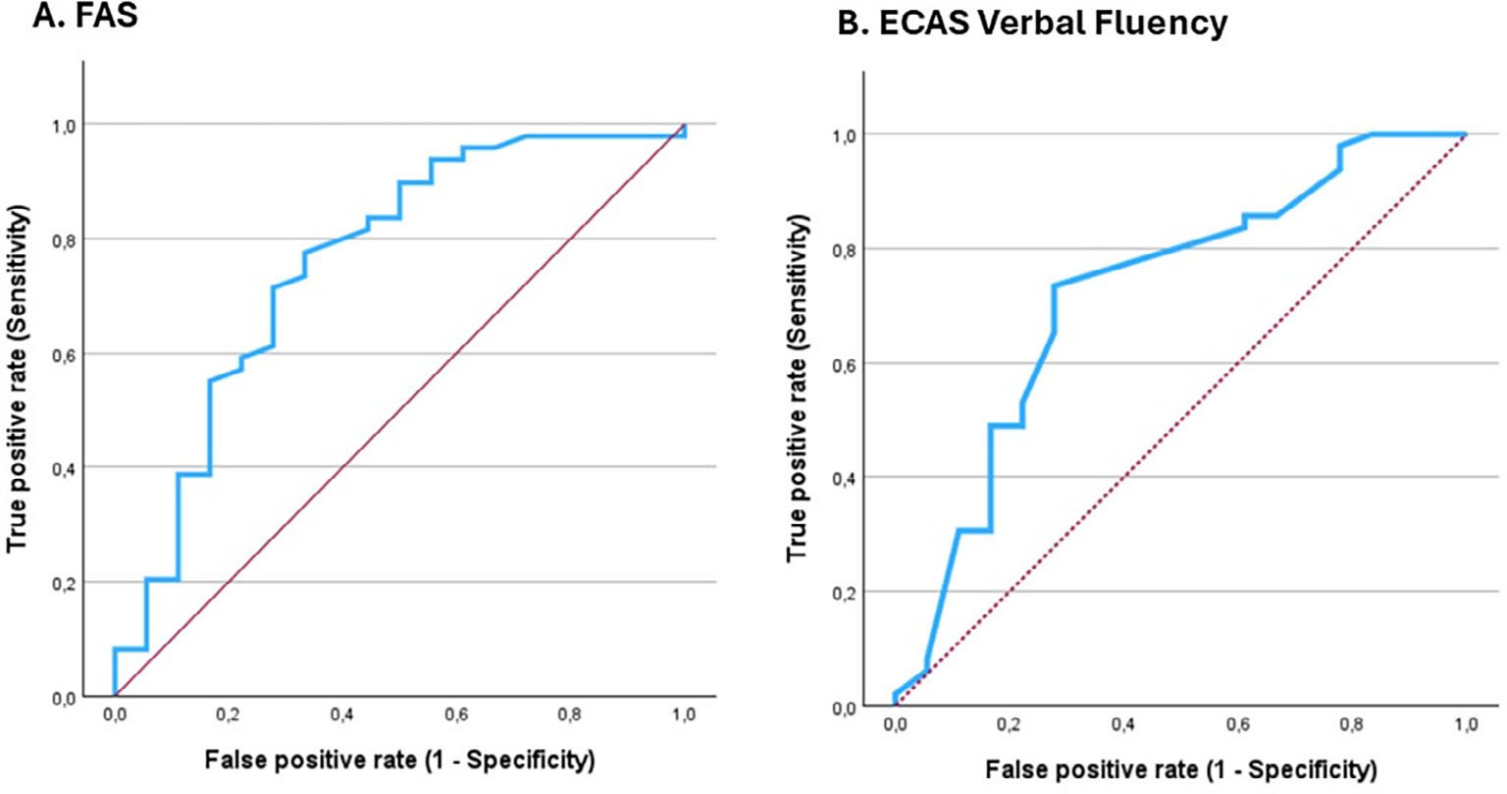
ALS‐CN patients. (A) Receiver operating characteristic (ROC) curve for FAS. (B) ROC curve for ECAS verbal fluency. FAS = Letter Fluency Test; ECAS = Edinburgh Cognitive and Behavioural ALS Screen. [Color figure can be viewed at www.annalsofneurology.org]

For ALS‐CN patients who phenoconverted, we calculated the receiver operating characteristic (ROC) curves of FAS and ECAS verbal fluency score. For FAS, we found an area‐under‐curve (AUC) of 0.759 (95% CI, 0.618–0.899), with 28.75 as the optimal cut‐off score for predicting subsequent cognitive impairment (sensitivity: 0.776, specificity: 0.333) according to Youden index (Fig 2A). For ECAS verbal fluency score, we found an AUC of 0.664 (95% CI, 0.543–0.784), with 14.2 as the optimal cut‐off score for predicting subsequent cognitive impairment (sensitivity: 0.703, specificity: 0.269) (see Fig 2B).

Interestingly, ALS‐CN patients who phenoconverted exhibited a higher ∆ALSFRSr between T0 and T1 (phenoconverters: median 0.64 [IQR: 0.44–1.10] vs. non‐phenoconverters: median 0.51 [IQR 0.14–0.79], *p* = 0.031) and had a shorter survival from T1 compared with non‐phenoconverters (see Fig S2).

### ALSbi, ALSci, and ALScbi Phenoconverters

Among the 22 ALSci patients, 8 (36.4%) phenoconverted to either ALScbi (5 cases) or ALS‐FTD (3 cases); 2 (16.7%) of 12 ALSbi patients phenoconverted to ALScbi, and 3 (50%) of 6 ALScbi patients phenoconverted to ALS‐FTD (see Fig 1B). Although ALSbi patients at T0 showed less cognitive decline than the other groups, this difference was not statistically significant. The 13 patients with cognitive worsening had significantly lower baseline scores in FAS (*p* = 0.02) and ECAS verbal fluency (*p* = 0.023). Line graphs of individual FAS and ECAS verbal fluency changes between T0 and T1 are shown in Figure S4. ROC curves for FAS and ECAS verbal fluency predicted cognitive decline. For FAS, the AUC was 0.735 (95% CI, 0.563–0.907) with a cut‐off of 22.65 (sensitivity: 0.885, specificity: 0.429) (Fig 4A). For ECAS verbal fluency, the AUC was 0.657 (95% CI, 0.463–0.850) with a cut‐off of 11.8 (sensitivity: 0.885, specificity: 0.571) (see Fig 4B).

### Results of [18F]FDG PET Imaging

**FIGURE 3:**
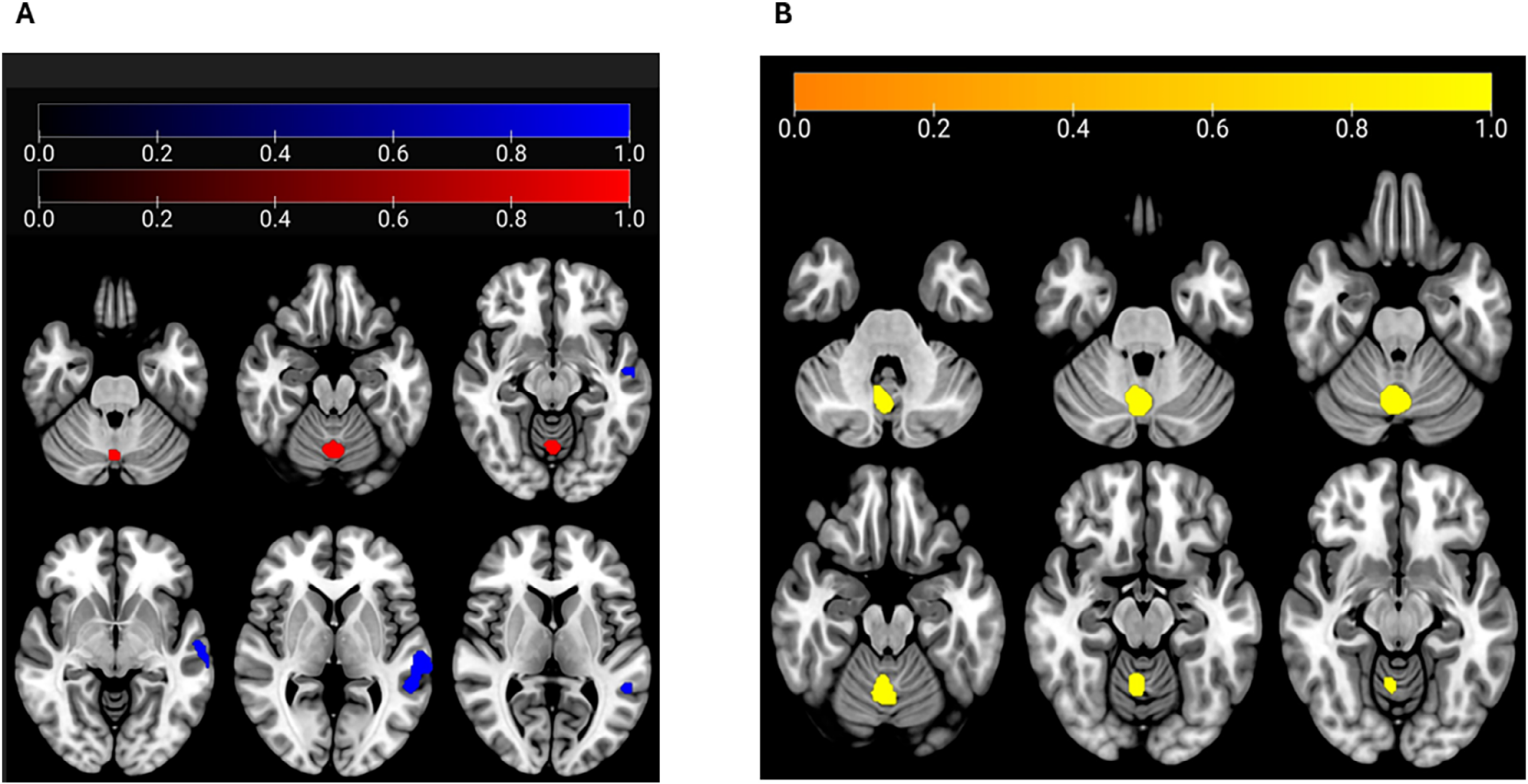
(A) The regions showing a statistically significant relative hypometabolism in amyotrophic lateral sclerosis patients with normal cognition (ALS‐CN) converters compared with ALS‐CN non‐converters are marked in blue. The regions showing a statistically significant relative hypermetabolism in ALS‐CN converters compared with ALS‐CN non‐converters are marked in red. (B) Clusters showing statistically significant relative hypermetabolism in ALS who worsened from T0 to T1 compared with those who did not are marked in yellow.

ALS‐CN who later phenoconverted showed relative hypometabolism at left temporal lobe (Broadman's areas 21 and 22) and relative hypermetabolism at the left cerebellar declive (Fig 3A and Table S5). The patients who were already cognitively impaired and experienced cognitive worsening between T0 and T1 exhibited relative hypermetabolism in a cerebellar cluster, including the right cerebellum, posterior lobe, and declive of the vermis, compared to those who did not worsen (see Fig 3B and Tables S6 and S7).

**FIGURE 4:**
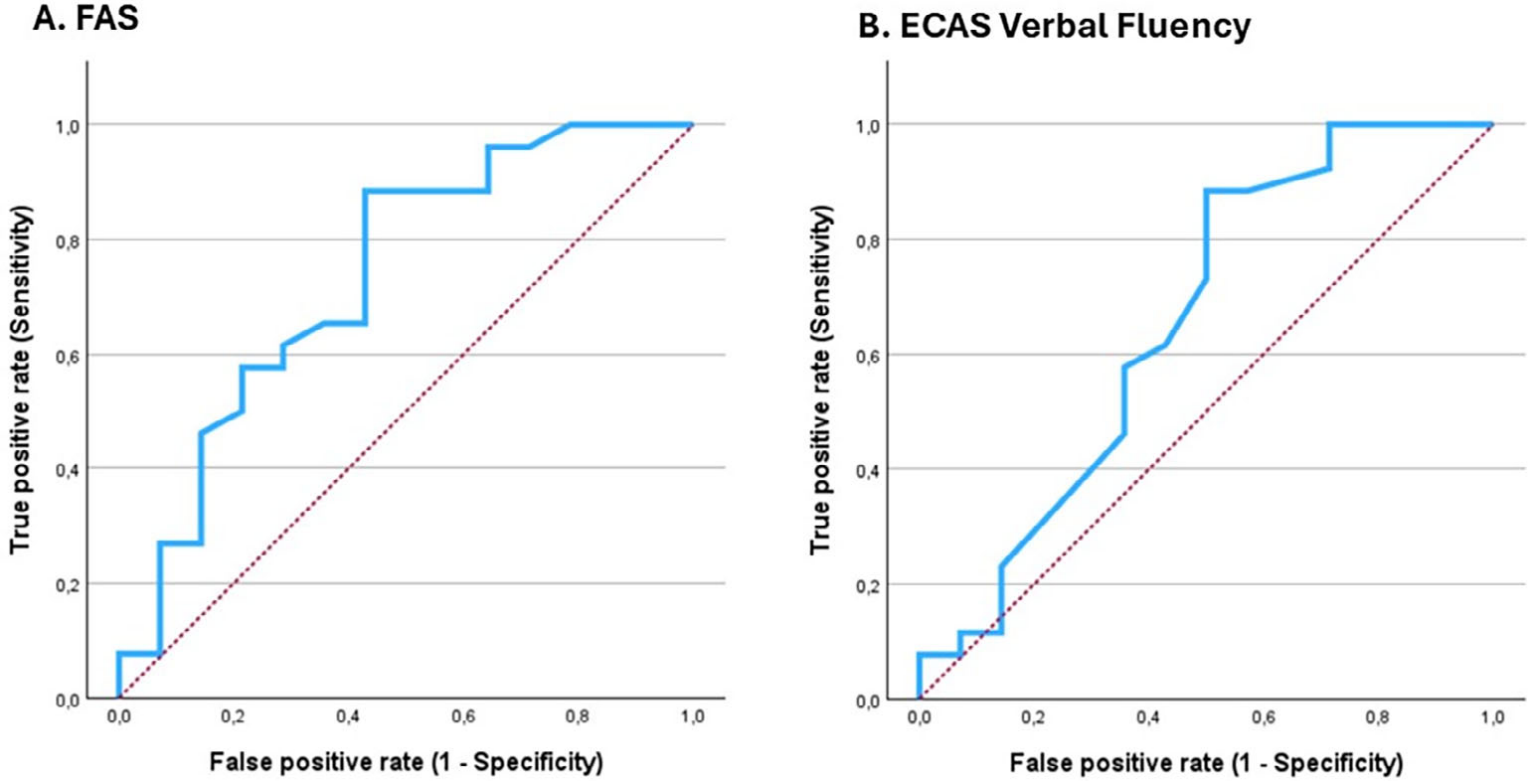
Amyotrophic lateral sclerosis (ALS) patients with isolated behavioral impairment, ALS patients with isolated cognitive impairment, and ALS patients with both cognitive and behavioral impairment. (A) Receiver operating characteristic (ROC) curve for FAS. (B) ROC curve for ECAS verbal fluency. FAS = Letter Fluency Test; ECAS = Edinburgh Cognitive and Behavioural ALS Screen. [Color figure can be viewed at www.annalsofneurology.org]

## Discussion

In this longitudinal cohort, more than 25% of ALS‐CN experienced cognitive phenoconversion, and 33% of patients who were already cognitively impaired experienced further cognitive deterioration within 1 year. Among patients cognitively normal at T0, the most predictive tests for phenoconversion were the FAS and the ECAS verbal fluency score. Other factors associated with phenoconversion included age, education, and the presence of genetic variants. Thirteen (32.5%) patients who were already cognitively impaired at T0 showed worsening of their cognitive status at T1. Only FAS and ECAS verbal fluency score were significantly lower at T0 in these patients.

The cognitive and behavioral changes in ALS patients have been examined in several longitudinal studies, with mixed results.^6^, ^9^, ^17^, ^20^, ^21^, ^22^ In the first comprehensive study assessing ALS cognition longitudinally, cognitively impaired patients at T0 experienced further cognitive decline, whereas those with normal baseline cognition generally remained stable.^6^ However, the high attrition rate of the study (75% of patients were not tested at 1 year) made it challenging to draw definitive conclusions. Another study observed behavioral changes over 12 months, but did not find significant cognitive deterioration.^9^ In an Italian longitudinal study based on a comprehensive series of cognitive tests, one‐third of patients showed worsening of cognitive function after a median time of 7 months: 88% of those with ALSbi, 27% of those with ALSci, 40% of those with ALScbi. Notably, also 24% of cognitively normal patients developed significant cognitive dysfunction.^20^ Cognitive decline was associated with older age at onset and lower education, although other factors were not explored. A Greek study found a decline in Montreal Cognitive Assessment and Frontal Assessment Battery scores over time in a longitudinal series of ALS patients, although their cognitive classification was not reported.^21^ Another study, which followed patients for 6 to 24 months, found a statistically significant decrease in ECAS ALS non‐specific scores, whereas ALS‐specific and total scores did not change.^17^ This study involved a relatively young and slow‐progressing cohort of patients, potentially introducing selection bias. Nonetheless, some patients, particularly *C9ORF72* repeat expansion carriers and those with lower education did show cognitive decline. Finally, a recent Swedish study noted that 20% of patients with normal baseline cognition experienced a decline over time, as measured by ECAS.^22^

In our series, we were able to re‐test approximately two‐thirds of patients after 1 year, nearly double the proportion seen in previous longitudinal studies. As expected, patients who were not re‐testable were older, had a lower ALSFRSr score, and a more severe King's staging.

Consistent with other studies, factors related to cognitive and behavioral deterioration included older age and lower education level, a proxy of cognitive reserve.^14^, ^18^, ^19^, ^34^ Of note, genetic burden, including but not limited to *C9ORF72* repeat expansion, emerged as a strong driver of cognitive deterioration, with an Odds Ratio (OR) >5, consistent with previous studies.^14^, ^16^, ^17^, ^35^ This highlights the significant role of genetics in shaping the ALS phenotype. However, also because of the limited sample of this study, we could not identify specific associations between the presence of genetic variants and cognitive deterioration.

One aim of this study was to identify early markers of cognitive deterioration. We found that phenoconversion from ALS‐CN, as well the worsening of cognitive status in patients who were already impaired at baseline, was associated with lower, yet not pathological, scores at T0 on FAS and ECAS verbal fluency, that is, both tests of phonemic fluency included in our battery. The binary logistic regression model, which allows correcting for known clinical factors related to cognitive impairment, confirmed that both FAS and ECAS verbal fluency scores were independently linked to phenoconversion. ROC curve analysis provided the optimal cut‐off scores of each test: 28.75 for FAS and 14.2 for ECAS verbal fluency, with sensitivities of approximately 75% and specificities of approximately 30%. Similar results were observed for deterioration in subjects who were already cognitively impaired, although, in this case, the predictive role of ECAS verbal fluency was borderline.

The strong role of phonemic fluency in diagnosing cognitive impairment in ALS aligns with the ALSFTD‐CC criteria,^26^ which note that impaired verbal fluency alone is sufficient to identify executive dysfunction. Previous studies have also highlighted that the verbal fluency score is the only ECAS subscore independently associated with more advanced King's stages of ALS^7^ and that it is particularly correlated with cognitive decline in patients with bulbar onset.^21^ The high predictive value of FAS or ECAS verbal fluency for future cognitive decline likely stems from its engagement with multiple cognitive domains, including executive functions—such as initiation, set‐shifting, sustained attention, and inhibition—as well as language abilities, particularly word retrieval.^36^, ^37^, ^38^

In other neurodegenerative disorders, phonemic fluency has been identified as an early marker of cognitive deterioration. For instance, it is relevant in Parkinson's disease (PD),^39^ Huntington's disease (HD),^40^ asymptomatic gene carriers for HD,^41^ Alzheimer's disease (AD), and preclinical AD.^42^, ^43^ However, in PD and AD semantic fluency is generally more impaired than phonemic fluency.^39^, ^42^ Notably, in our study, semantic fluency did not emerge as a predictor of future cognitive dysfunction.

In our cohort, cognitive and behavioral worsening in patients who were ALS‐CN at T0 was related to more rapid disease as measured by ∆ALSFRSr and also shorter survival. This finding is consistent with previous longitudinal^6^, ^20^, ^21^ and cross‐sectional studies.^7^, ^12^ Additionally, although not statistically significant, there was a tendency toward a higher increase in both King's and MiToS staging.

The [18F]FDG‐PET study in a subset of patients ALS‐CN showed a significant hypometabolism in left temporal areas among those who phenoconverted. Although phonemic fluency is typically associated with left frontoparietal regions, several magnetic resonance imaging studies have also highlighted the role of left temporal brain regions.^44^, ^45^, ^46^ The prefrontal cortex primarily supports the executive functions necessary for tasks like FAS, whereas the temporal lobe contributes through its involvement in word retrieval, lexical storage, and phonological processing. The extended involvement of brain regions in phenoconverters is further corroborated by their lower scores on tests of verbal memory (RAVL‐IR), visuoconstructive abilities (ROCF‐IR), and nonverbal intelligence (CPM47). When analyzing all cognitive categories together, we observed similar findings in patients who exhibited cognitive or behavioral worsening at T1 compared with those who did not. We also found relative hypermetabolism in the median cerebellar region. Previous studies have linked cerebellar hypermetabolism to astrocytosis or microglial activation.^47^, ^48^ Additionally, given the cerebellum's role in modulating the neural circuitry linking the prefrontal, posterior parietal, superior temporal, and limbic cortices, it is plausible that in the presence of cognitive involvement, compensatory changes in the cerebellum are more prominent, acting as an adaptive mechanism to mitigate frontotemporal cognitive impairment.^49^

This study has some limitations. First, approximately 35% of the patients originally enrolled could not be re‐tested after 1 year because of clinical deterioration, a common challenge of longitudinal ALS studies.^6^, ^22^ Possible solutions include implementing more frequent sampling or using gaze‐controlled versions of cognitive tests. Our analysis of factors contributing to attrition suggests that the inability to complete testing in these non‐retested patients may have led to an underestimation of phenoconverters. Second, social cognition tests were administered to only a subset of patients, which limits our ability to thoroughly assess this emerging domain of cognitive function in ALS. Despite these limitations, the study's strengths include the use of a comprehensive battery of cognitive tests alongside the ECAS, enhancing our understanding of cognitive deterioration in ALS. Additionally, the population‐based nature of the study helps mitigate selection bias. Third, we have not performed a longitudinal evaluation of HCs. However, this evaluation is outside the scope of this study, because HCs were recruited only to establish an internal cut‐off score for cognitive tests, in addition to the Italian normative, and not to study the modification of tests over time. Fourth, a common challenge in longitudinal studies on cognitive function is the potential learning effect, that is, improvements in test performance because of increased familiarity with the test instruments, paradigms, and items.^50^ This issue can obscure potential cognitive decline and remains a significant concern in both clinical and research settings. To address this, we re‐administered the test battery after a 1‐year interval. In our cohort, however, approximately one‐fourth of patients showed a slight increase in either the FAS score or the ECAS verbal fluency score from T0 to T1. Consequently, the learning effect observed may have led to an underestimation of cognitive and behavioral deterioration in some patients.

In conclusion, cognitive and behavioral functions in ALS are not stable over time for a significant subset of patients, including those who are cognitively normal at the time of diagnosis. Factors such as older age, lower education levels, and the presence of genetic variants contribute to cognitive deterioration. Tests assessing phonemic fluency serve as early predictors of future cognitive decline, and we propose cut‐off scores to identify individuals at risk. Given the substantial impact of cognitive and behavioral impairments on clinical progression and quality of life for both patients and caregivers, early detection of those at risk and the need for longitudinal cognitive assessments are essential, particularly in managing end‐of‐life care. Further research is needed to explore additional predictors of cognitive phenoconversion. Nonetheless, our findings highlight the role of phonemic fluency not only as an early marker of cognitive impairment, but also as a predictor of cognitive decline during the course of the disease.

## Author Contributions

B.I., F.P., C.M., G.M., A.Can., A.Cal., and A.Ch. contributed to the conception and design of the study; B.I., F.P., C.M., G.M., U.M., M.G., E.M., S.C., M.B., R.V., M.P., A.Can., A.Cal., and A.Ch. contributed to the acquisition and analysis of data; B.I., G.M., A.Can., A.Cal., and A.Ch. contributed to drafting the text or preparing the figures.

## Potential Conflicts of Interest

Nothing to report.

## Supporting information


**Data S1.** Supporting Information.

## Data Availability

Anonymized data relating to this article will be made available by request from any qualified investigator, subject to approval from the Comitato Etico Azienda Ospedaliero‐Universitaria Città della Salute e della Scienza.
