# Supplementary material for: Frequency and Early Predictors of Cognitive Deterioration in Amyotrophic Lateral Sclerosis: A Longitudinal Population‐Based Study
Source: Ann Neurol. 2025 Feb 1;97(6):1122–33. doi: 10.1002/ana.27194 (PMC12082020; doi:10.1002/ana.27194)

**Supplementary methods**

***List of performed tests***. Executive function was tested with Letter Fluency test (FAS), Category Fluency Test (CAT), Trail Making Test B-A (TMT B-A), Frontal Assessment Battery (FAB), ECAS Executive Function score, and ECAS Verbal Fluency score; Language was assessed with ECAS Language subscore and Boston Naming Test; Verbal Memory with Rey Auditory Verbal Learning Test, Immediate Recall (RAVL-IR), Rey Auditory Verbal Learning Test, delayed Recall (RAVL-DR), Babcock Story Recall Test, Immediate Recall (BSRT-IR), Babcock Story Recall Test, Delayed Recall (BSRT-DR), and ECAS Memory score; Visual Memory with Rey-Osterrieth Complex Figure Test, differed recall (ROCF-DR); Visuoconstructive abilities with Rey-Osterrieth Complex Figure Test, Immediate Recall (ROCF-IR), Clock Drawing Test (Clock), and ECAS Visuospatial Abilities score; Attention/Working memory with Digit Span Forward (FW) and Digit Span Backward (BW); Psychomotor speed with Trail Making Test A (TMT A); Non-Verbal Intelligence with Raven’s Colored Progressive Matrices (CPM47); Cognitive flexibility with Trail Making Test B (TMT B); Theory of Mind (social cognition) with Story-based Empathy Task (SET); Behavior with Frontal Systems Behavior Scale (FrSBe), ECAS, and Frontal Behavioural Inventory (FBI). For the Frontal Systems Behaviour Scale (FrSBe), using the Family-form evaluated by a close relative/caregiver (scores: normal ≤59, borderline 60-64; pathological ≥65). For the purpose of this study, we considered the change in points for each of the 3 domains of FrSBe (apathy, disinhibition, executive) from the before disease to the disease scores. If a subject had scores reflecting a frontal systems abnormality both in the premorbid and in the post-illness forms, he/she was considered pathological only if there was an increase of ≥10 points at the T-score between the two forms (Montuschi et al, 2015).

For ECAS we used the validated Italian version (Poletti et al 2018), and for the behavioral component of ECAS the cut-off based on number of symptoms (Poletti et al 2023).

**Supplementary references**

Montuschi A, Iazzolino B, Calvo A, Moglia C, Lopiano L, Restagno G, Brunetti M, Ossola I, Lo Presti A, Cammarosano S, Canosa A, Chiò A. Cognitive correlates in amyotrophic lateral sclerosis: a population-based study in Italy. J Neurol Neurosurg Psychiatry. 2015 Feb;86(2):168-73. doi: 10.1136/jnnp-2013-307223. PMID: 24769471.

Poletti B, Solca F, Carelli L, Madotto F, Lafronza A, Faini A, Monti A, Zago S, Calini D, Tiloca C, Doretti A, Verde F, Ratti A, Ticozzi N, Abrahams S, Silani V. The validation of the Italian Edinburgh Cognitive and Behavioural ALS Screen (ECAS). Amyotroph Lateral Scler Frontotemporal Degener. 2016 Oct-Nov;17(7-8):489-498. doi: 10.1080/21678421.2016.1183679. Epub 2016 May 24. PMID: 27219526.

Poletti B, Aiello EN, Solca F, Torre S, Carelli L, Ferrucci R, Verde F, Ticozzi N, Silani V. Diagnostic properties of the Italian ECAS Carer Interview (ECAS-CI). Neurol Sci. 2023 Mar;44(3):941-946. doi: 10.1007/s10072-022-06505-x. PMID: 36417015.

**Supplementary Table 1**. Cognitive tests: explored domains

| **Domains** | **Tests** |
| --- | --- |
| Executive functions | Letter Fluency test (FAS) |
|  | Category Fluency Test (CAT) |
|  | Trail Making Test B-A (TMT B-A) |
|  | Frontal Assessment Battery (FAB) |
|  | ECAS Executive Function score |
|  | ECAS Verbal Fluency score |
| Verbal memory | Rey Auditory Verbal Learning Test, Immediate Recall (RAVL-IR) |
|  | Rey Auditory Verbal Learning Test, delayed Recall (RAVL-DR) |
|  | Babcock Story Recall Test, Immediate Recall (BSRT-IR) |
|  | Babcock Story Recall Test, Delayed Recall (BSRT-DR) |
|  | ECAS Memory score |
| Language | Token test (up to 2016) |
|  | Battery for the Analysis of Aphasic Deficits (semantic systems tests 7 and 8) (up to 2016) |
|  | Boston Naming Test (from 2016) |
|  | ECAS Language score (from 2016) |
| Visual Memory | Rey-Osterrieth Complex Figure Test, differed recall (ROCF-DR) |
| Visuoconstructive abilities | Rey-Osterrieth Complex Figure Test, Immediate Recall (ROCF-IR) |
|  | Clock Drawing Test (Clock) |
|  | ECAS Visuospatial Abilities score |
| Attention/working memory | Digit Span Forward (FW) |
|  | Digit Span Backward (BW) |
| Psychomotor speed | Trail Making Test A (TMT A) |
| Fluid intelligence | Raven’s Colored Progressive Matrices (CPM47) |
| Cognitive flexibility | Trail Making Test B (TMT B) |
| Theory of mind (social cognition) | Story-based Empathy Task (SET) (from 2018) |
| Behavior | Frontal Systems Behavior Scale (FrSBe) |
|  | ECAS Behavior score |
|  | Frontal Behavioural Inventory (FBI) |
| Non-ALS | Mini Mental State Examination (MMSE) |

**Supplementary Table 2**. References of normative data of used tests

| **Test** | **Italian normative reference** |
| --- | --- |
| MMSE | Carpinelli Mazzi M, Iavarone A, Russo G, et al. Mini-Mental State Examination: new normative values on subjects in Southern Italy. Aging Clin Exp Res. 2020;32(4):699-702. doi:10.1007/s40520-019-01250-2 |
| FAS | Caltagirone C, Gainotti G, Carlesimo GA, et al. Batteria per la valutazione del Deterioramento Mentale (parte I): descrizione di uno strumento di diagnosi neuropsicologica. Archivio Di Psicologia, Neurologia E Psichiatria 1995; 56(4), 461-470 |
| CAT | Spinnler H, Tognoni G. Standardizzazione e taratura italiana di test neuropsicologici. Ital J Neurol Sci. 1987; 6 [Suppl. 8]: 78-80 |
| FAB | Appollonio I, Leone M, Isella V, et al. The frontal assessment battery (FAB): Normative values in an Italian population sample. Neurol Sci. 2005;26(2):108-116. doi:10.1007/s10072-005-0443-4 |
| Digit Span FW and BW | Monaco M, Costa A, Caltagirone C, Carlesimo GA. Forward and backward span for verbal and visuo-spatial data: Standardization and normative data from an Italian adult population. Neurol Sci. 2013;34(5):749-754. doi:10.1007/s10072-012-1130-x |
| TMT A, B and B-A | Siciliano M, Chiorri C, Battini V, Sant'Elia V, Altieri M,v Trojano L, Santangelo G. Regression-based normative data and equivalent scores for Trail Making Test (TMT): an updated Italian normative study. Neurol Sci. 2019 Mar;40(3):469-477. doi: 10.1007/s10072-018-3673-y. |
| RAVL-IR and IR | Caltagirone C, Gainotti G, Carlesimo GA, et al. Batteria per la valutazione del Deterioramento Mentale (parte I): descrizione di uno strumento di diagnosi neuropsicologica. Archivio di Psicologia, Neurologia e Psichiatria 1995; 56(4), 461-470 |
| BSRT-IR and DR | Carlesimo GA, Buccione I, Fadda L, et al. Normative data of two memory tasks: Short-Story recall and Rey’s Figure. Nuova Riv di Neurol 2002;12(1):1-13. |
| ROCF-IR and DR | Carlesimo GA, Buccione I, Fadda L, et al. Normative data of two memory tasks: Short-Story recall and Rey’s Figure. Nuova Riv di Neurol 2002;12(1):1-13. |
| CPM47 | Caltagirone C, Gainotti G, Carlesimo GA, et al. Batteria per la valutazione del Deterioramento Mentale (parte I): descrizione di uno strumento di diagnosi neuropsicologica. Archivio di Psicologia, Neurologia e Psichiatria 1995; 56(4), 461-470 |
| ECAS | Poletti B, Solca F, Carelli L, Madotto F, Lafronza A, Faini A, Monti A, Zago S, Calini D, Tiloca C, Doretti A, Verde F, Ratti A, Ticozzi N, Abrahams S, Silani V. The validation of the Italian Edinburgh Cognitive and Behavioural ALS Screen (ECAS). Amyotroph Lateral Scler Frontotemporal Degener. 2016 Oct-Nov;17(7-8):489-498. doi: 10.1080/21678421.2016.1183679. |
| SET | Dodich A, Cerami C, Canessa N, Crespi C, Iannaccone S, Marcone A, Realmuto S, Lettieri G, Perani D, Cappa SF. A novel task assessing intention and emotion attribution: Italian standardization and normative data of the Story-based Empathy Task. Neurol Sci. 2015 Oct;36(10):1907-12. doi: 10.1007/s10072-015-2281-3. Epub 2015 Jun 14. PMID: 26072203. |

**Supplementary Table 3.** Cognitive classification of ALS patients enrolled in the study who were and were not reassessed at T1 (p=0.0001).

|  | **Reassessed patients**  **(N=107)** | **Non-reassessed patients (n=54)** | **Patients originally enrolled (N=161)** |
| --- | --- | --- | --- |
| ALS-CN | 67 (62.6%) | 16 (29.6%) | 83 (51.6%) |
| ALSbi | 12 (11.2%) | 1 (1.9%) | 13 (8.1%) |
| ALSci | 22 (20.6%) | 21 (38.9%) | 43 (26.7%) |
| ALScbi | 6 (5.6%) | 6 (11.1%) | 12 (7.5%) |
| ALS-FTD | 0 | 10 (18.5%) | 10 (6.2%) |

ALSbi, patients with isolated behavioral impairment; ALScbi, patients with both cognitive and behavioral impairment; ALSci, patients with isolated cognitive impairment; ALS-CN, patients with normal cognition; ALS FTD, patients with frontotemporal dementia; WT, ALS patients not carrying pathogenic mutations

**Supplementary Table 4**. List of genetic variants identified in the study cohort and the corresponding cognitive/behavioral classification

| **Gene** | **# of cases** | **Cognitive/behavioral classification** | |
| --- | --- | --- | --- |
|  |  | **T0** | **T1** |
| *C9ORF72* | 8 | ALS-CN (3), ALSci (3), ALSbi (2) | ALS-CN (2), ALSci (2), ALSbi (1), ALScbi (1), ALS-FTD (2) |
| *C9ORF72 & TARDBP* | 1 | ALSbi | ALSbi |
| *DCTN1* | 1 | ALS-CN | ALS-FTD |
| *KIF5A* | 2 | ALS-CN (2) | ALS-CN (1), ALScbi (1) |
| *MATR3* | 2 | ALS-CN (1), ALSci (1) | ALS-CN (1), ALSci (1) |
| *NEK1* | 1 | ALS-CN | ALS-CN |
| *OPTN* | 1 | ALSbi | ALSbi |
| *PFN1* | 1 | ALS-CN | ALScbi |
| *SETX* | 1 | ALS-CN | ALS-CN |
| *SIGMAR1* | 1 | ALSci | ALSci |
| *SOD1* | 2 | ALS-CN, ALSbi | ALS-CN, ALSbi |
| *SQSTM1* | 1 | ALS-CN | ALS-CN |
| *TARDBP* | 4 | ALS-CN (3), ALSci (1) | ALS-CN (3), ALSci (1) |
| *TBK1* | 3 | ALS-CN (3) | ALSci (1), ALScbi (2) |
| *FIF4* | 1 | ALS-CN | ALSci |
| WT | 77 | ALS-CN (49), ALSci (16), ALSbi (7), ALScbi (6) | ALS-CN (38), ALSci (13), ALSbi (9), ALScbi (13), ALS-FTD (4) |

**Supplementary Table 5**. Comparison of tests performances at T0 of CN-ALS patients who had a cognitive/behavioral phenoconversion at T1 and those who did not phenoconverted

|  | **ALS-CN phenoconverters**  **n=18** | **ALS-CN non- phenoconverters**  **n=49** | **p-value** |
| --- | --- | --- | --- |
| MMSE | 27.6 (26.3-29.6)  N=18 | 29.0 (27.0-30.0)  N=49 | 0.119 |
| FAS | 26.5 (21.0-31.9)  N=18 | 33.0 (28.9-36.3)  N=49 | **0.001** |
| CAT | 21.8 (16.9-22.4)  N=18 | 19.8 (16.8-24.8)  N=49 | 0.763 |
| FAB | 15.1 (14.5-16.1)  N=18 | 15.2 (14.2-16.7)  N=49 | 0.829 |
| Digit Span FW | 5.7 (5.0-6.3)  N=18 | 5.9 (5.4-6.5)  N=46 | 0.582 |
| Digit Span BW | 3.8 (3.4-4.3)  N=18 | 4.0 (3.5-4.8)  N=46 | 0.293 |
| TMT A | 36.0 (21.8-46.8)  N=18 | 30 (20.5-43)  N=46 | 0.350 |
| TMT B | 54 (34-103)  N=18 | 55 (40-90)  N=46 | 0.840 |
| TMT B-A | 38 (7-84)  N=18 | 27.5 (7-55)  N=46 | 0.938 |
| RAVL-IR | 39 (36-44.2)  N=18 | 46.9 (39.5-52.3)  N=45 | **0.019** |
| RAVL-DR | 8.2 (6.1-10)  N=18 | 9.8 (7.62-11.4)  N=45 | 0.088 |
| BSRT-IR | 6.8 (5.4-7.5)  N=13 | 6.1 (5 -7)  N=40 | 0.277 |
| BSRT-DR | 7.4 (6.2-8.0)  N=13 | 7.1 (5.9-8.0)  N=40 | 0.559 |
| ROCF-IR | 30.8 (28.5-32)  N=17 | 32.5 (30.4-34)  N=40 | **0.043** |
| ROCF-DR | 12.5 (7.9-14.3)  N=17 | 11.4 (8.3-18.3)  N=40 | 1.000 |
| Clock | 5 (3-5)  N=18 | 5 (4-5)  N=49 | 0.255 |
| CPM47 | 28.3 (24.9-31.6)  N=18 | 30.3 (28.4-33.1)  N=47 | **0.021** |
| SET Intention Attribution | 6 (5-7)  N=10 | 5.2 (4.8-6)  N=18 | 0.469 |
| SET Causal Inference | 6 (3-7)  N=10 | 4.9 (3.8-5.2)  N=18 | 0.371 |
| SET Emotion Attribution | 5 (2-6)  N=10 | 4.1 (3.8-5.4)  N=18 | 0.811 |
| SET Global Score | 17 (11-20)  N=10 | 13.7 (12-16.1)  N=18 | 0.573 |
| HADS-A | 7 (4-11)  N=18 | 7.5 (5-9)  N=49 | 0.814 |
| HADS-D | 5 (2-6)  N=18 | 4 (2-6.5)  N=49 | 0.823 |
| ECAS Language | 26 (25-27)  N=18 | 27 (24.5-28)  N=43 | 0.260 |
| ECAS Verbal Fluency | 14 (11.5-16.4)  N=18 | 17.5 (14-20)  N=43 | **0.017** |
| ECAS Executive | 33 (30.5-38.5)  N=18 | 34.5 (30-37.8)  N=43 | 0.885 |
| ECAS Memory | 19 (16.5-21)  N=18 | 18 (16-20)  N=43 | 0.577 |
| ECAS Visuospatial | 12 (11-12)  N=18 | 12 (11-12)  N=43 | 0.529 |
| ECAS ALS specific score | 73 (67-80)  N=18 | 77 (69-85)  N=43 | 0.228 |
| ECAS non-ALS specific score | 30 (27.5-32.5)  N=18 | 30 (27-32)  N=43 | 0.698 |
| ECAS total score | 104 (94-112)  N=18 | 107 (97-114)  N=43 | 0.455 |

ALS-CN, ALS patients with normal cognition; BSRT, Babcock Story Recall Test; BW, backward; CAT, Category Fluency Test; Clock, Clock Drawing Test; CPM47, Raven’s Colored Progressive Matrices; ECAS, Edinburgh Cognitive and Behavioural ALS Screen FAB, Frontal Assessment Battery; DR, delayed recall; FAS, Letter Fluency test; FW, forward; HADS-A, Hospital Anxiety and Depression Scale- Anxiety; HADS-D, Hospital Anxiety and Depression Scale- Depression; IR, immediate recall; MMSE, Mini Mental State Examination; RAVL, Rey Auditory Verbal Learning Test; ROCF, Rey-Osterrieth Complex Figure Test; SET, Story-based Empathy Task; TMT, Trail Making Test

**Supplementary Table 6**. Clusters showing statistically significant relative hypometabolism and hypermetabolism in ALS-CN who phenoconverted compared to those who did not. BA: Brodmann Area.

| **Areas of relative hypometabolism** | | | | | | | |
| --- | --- | --- | --- | --- | --- | --- | --- |
| Cluster p  (FDR-corrected) | Cluster extent | Z-score | Talairach Coordinates | | | Cortical areas |  |
|  | | | x | y | z |  | BA |
| 0.036 | 404 | 3.94 | -63.0 | -25.0 | 1.0 | Left Superior Temporal Gyrus | 22 |
|  |  | 3.61 | -51.0 | -41.0 | 6.0 | Left Middle Temporal Gyrus | 22 |
|  |  | 3.31 | -57.0 | -14.0 | -6.0 | Left Middle Temporal Gyrus | 21 |
|  |  |  |  |  |  |  |  |
| **Areas of relative hypermetabolism** | | | | | | | |
| Cluster p  (FDR-corrected) | Cluster extent | Z-score | Talairach Coordinates | | | Cortical areas |  |
|  | | | x | y | z |  |  |
| 0.045 | 281 | 3.95 | 0.0 | -65.0 | -12.0 | Left Cerebellum Posterior Lobe | Declive |

**Supplementary Table 7**. Clusters showing statistically significant relative hypermetabolism in ALS who worsened from T0 to T1 compared to those who did not.

| Cluster p  (FDR-corrected) | Cluster extent | Z-score | Talairach Coordinates | | | Brain region |
| --- | --- | --- | --- | --- | --- | --- |
|  | | | x | y | z |  |
| 0.014 | 608 | 4.15 | 2.0 | -65.0 | -19.0 | Right Cerebellum, Posterior Lobe, Declive of Vermis |

**Supplementary Figure 1.** Survival from T0 comparing re-tested (blue line) and non-ri-tested patients (red line) (p<0.0001).Ticks indicate censored patients.

**
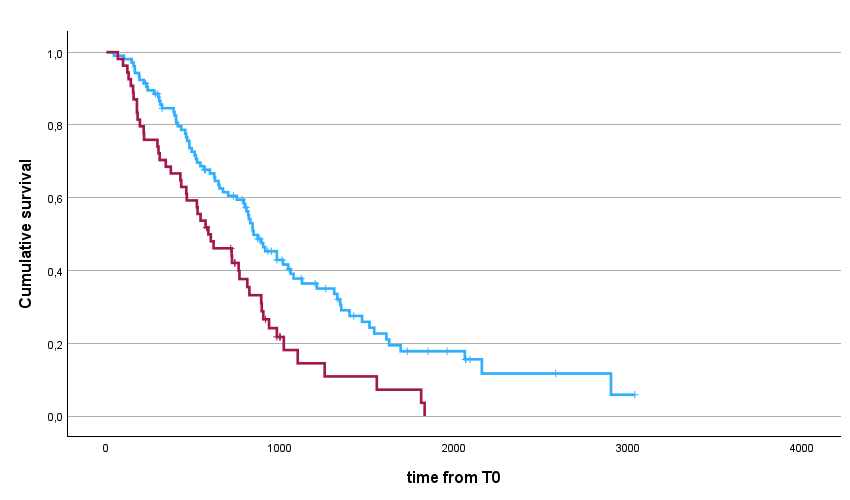
**

**Supplementary Figure 2**. Survival from T1 in AL-CN at T0 comparing patients who phenoconverted (red line) and those who did not (blue line) Phenoconverters: median survival time 624 days (IQR 299-1098). Non-phenoconverters median survival time1045 days (IQR 507-1541). p=0.026.Ticks indicate censored patients.


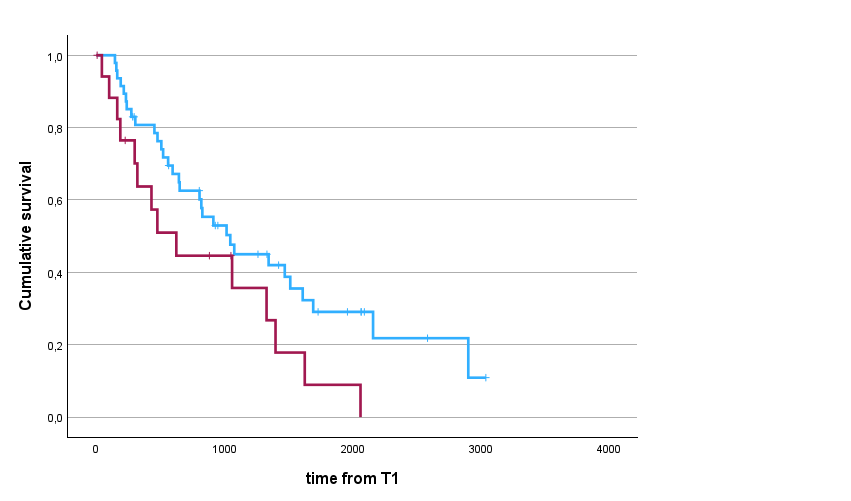


**Supplementary Figure 3**. Line graphs reporting individual changes of FAS score (A) and ECAS Verbal Fluency Score (B) in CN patients at T0 who did not phenoconvert (blue lines) and phenoconverted (red lines). Patients who phenoconverted to ALSbi are indicated with red dashed lines


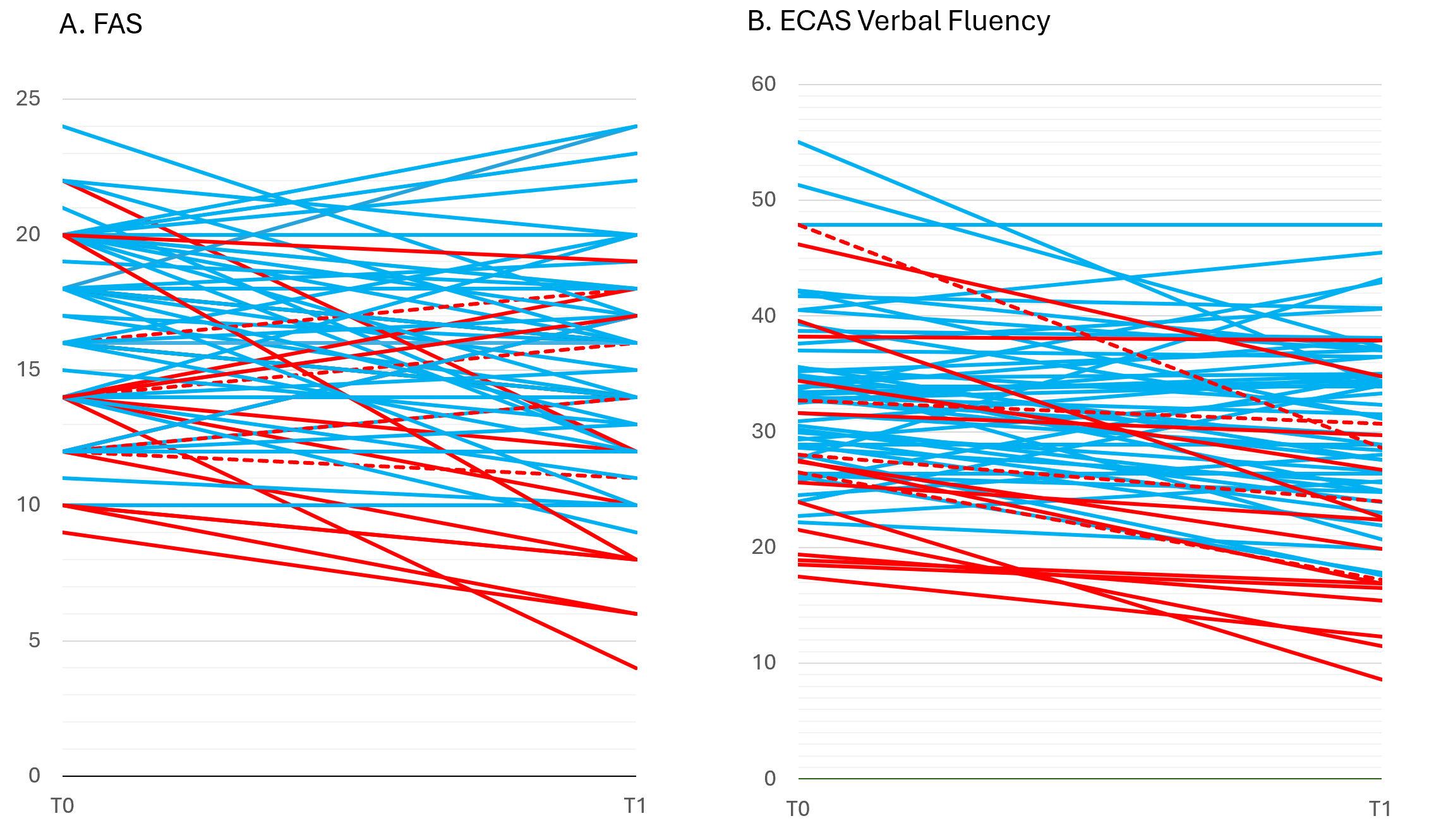


**Supplementary Figure 4**. Line graphs showing individual changes in FAS score (A) and ECAS Verbal Fluency score (B) over time in ALSci, ALSbi, and ALScbi patients at T0. Blue lines represent patients who did not phenoconvert, while red lines represent those who phenoconverted. Patients who phenoconverted to FTD are indicated by green lines. ALSbi patients who did not phenoconvert are indicated by blue dashed lines.


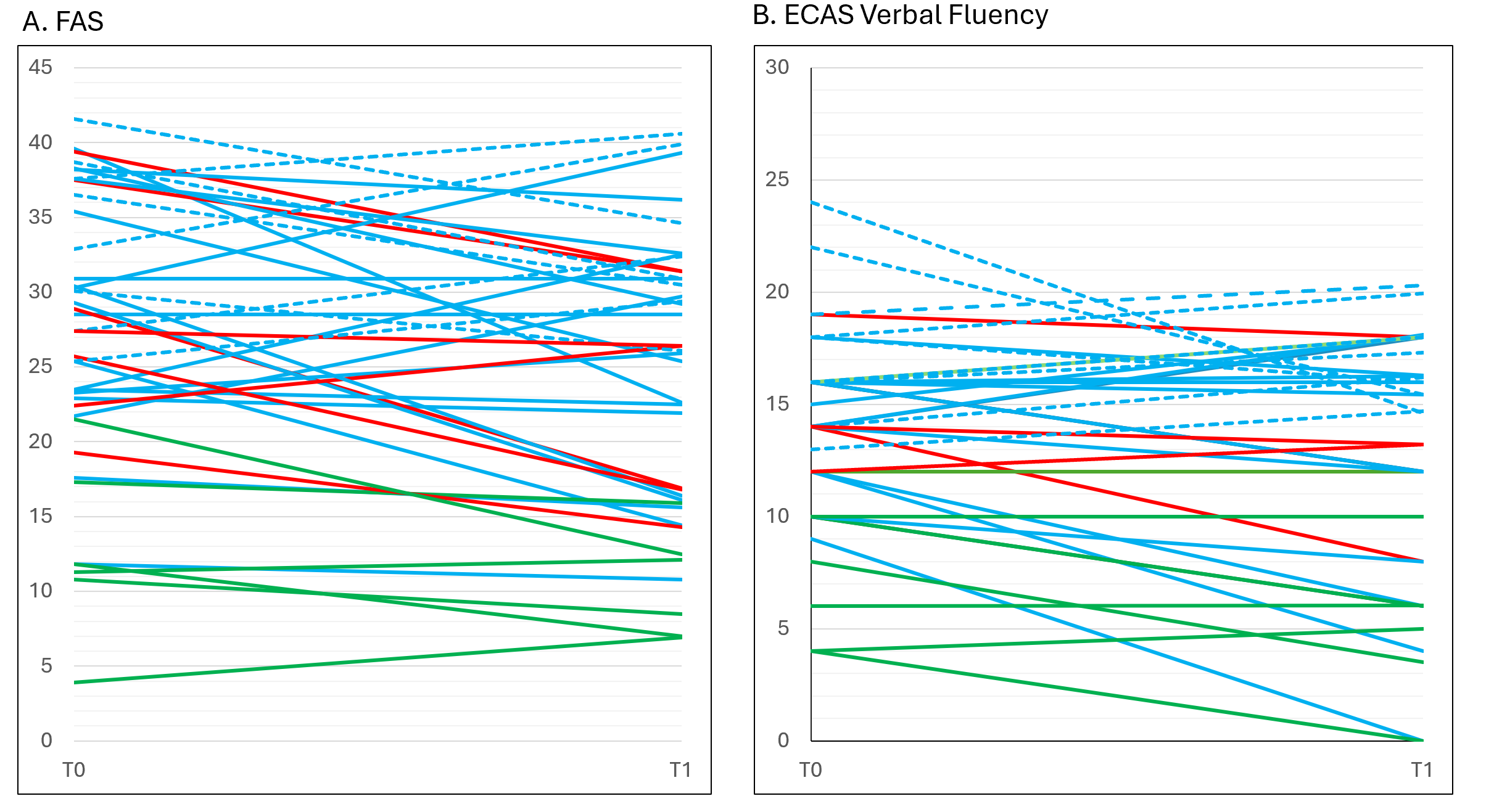

Supplement: Supplementary file 1 — Data S1. Supporting Information. [file ANA-97-1122-s001.docx]
